# A Qualitative Systematic Review of Effects of Provider Characteristics and Nonverbal Behavior on Pain, and Placebo and Nocebo Effects

**DOI:** 10.3389/fpsyt.2019.00242

**Published:** 2019-04-15

**Authors:** Hojjat Daniali, Magne Arve Flaten

**Affiliations:** ^1^Department of Humanity Sciences, Shahed University, Tehran, Iran; ^2^Department of Psychology, Norwegian University of Science and Technology, Trondheim, Norway

**Keywords:** contextual factors, experimenter characteristics, experimenter sex, clinician sex, nonverbal behavior, placebo effect, nocebo effect, pain

## Abstract

**Background:** Previous research has indicated that the sex, status, and nonverbal behaviors of experimenters or clinicians can contribute to reported pain, and placebo and nocebo effects in patients or research participants. However, no systematic review has been published.

**Objective:** The aim of this study was to investigate the effects of experimenter/clinician characteristics and nonverbal behavior on pain, placebo, and nocebo effects.

**Methods:** Using EmBase, Web of Knowledge, and PubMed databases, several literature searches were conducted to find studies that investigated the effects of the experimenter’s/clinician’s sex, status, and nonverbal behaviors on pain, placebo, and nocebo effects.

**Results:** Thirty-four studies were included, 20 on the effects of characteristics of the experimenter/clinician, 11 on the role of nonverbal behaviors, and 3 on the effects of both nonverbal behaviors and characteristics of experimenters/clinicians on pain and placebo/nocebo effects. There was a tendency for experimenters/clinicians to induce lower pain report in participants of the opposite sex. Furthermore, higher confidence, competence, and professionalism of experimenters/clinicians resulted in lower pain report and higher placebo effects, whereas lower status of experimenters/clinicians such as lower confidence, competence, and professionalism generated higher reported pain and lower placebo effects. Positive nonverbal behaviors (e.g., smiling, strong tone of voice, more eye contact, more leaning toward the patient/participant, and more body gestures) contributed to lower reported pain and higher placebo effects, whereas negative nonverbal behaviors (i.e., no smile, monotonous tone of voice, no eye contact, leaning backward from the participant/patient, and no body gestures) contributed to higher reported pain and nocebo effects.

**Conclusion:** Characteristics and nonverbal behaviors of experimenters/clinicians contribute to the elicitation and modulation of pain, placebo, and nocebo effects.

## Introduction

The present qualitative review investigated whether the characteristics or nonverbal behavior (NB) of the person administrating painful stimulation affected pain or placebo/nocebo effects in the research participant. The placebo effect is a psychobiological response that may occur following the application of active and inactive interventions (1). Applying an inactive medication paired with positive information about its analgesic effects can reduce pain (2). Likewise, negative information can reverse the analgesic effect of the medication (3, 4) and is called a “nocebo effect” (5, 6). Classical conditioning (previous experience with a treatment) and verbal information about the efficacy of the treatment are involved in the induction of placebo effects and expectations, that a treatment will reduce a symptom (e.g., pain), mediate the effects of both processes (7, 8).

Expecting that a procedure will increase pain may elicit anxiety and increase pain, whereas expecting that a procedure will decrease pain may reduce anxiety and thus reduce pain (9–12). As noted, placebo effects are induced by verbal information and/or classical conditioning [e.g., Refs. (2, 4, 12–14)]. However, other factors can modulate the experience of pain and placebo and nocebo effects. Treatments, whether active or sham, are administered in a compound of situational elements such as medication features (e.g., color of a tablet), the healthcare setting (hospital or clinic layout), and the characteristics and behavior of the experimenter/clinician. Such subtle cues in the environment (7, 15, 16) can affect the treatment outcome. For instance, Levine and Gordon (17) used three different methods of administering an inert substance (injection by a person sitting beside the patient and giving suggestive information; injection by a person in an adjacent room; or an injection by a programmable machine) and showed that even subtle cues that suggest a painkiller was administered could elicit a placebo response.

This systematic review is aimed to focus on the fields of pain and placebo/nocebo effects, due to their large literature background. This review is to our knowledge the first investigation of whether cues such as characteristics and NBs of the experimenter or clinician can affect pain, and placebo and nocebo effects.

### Experimenter/Clinician Characteristics

Characteristics of experimenters/clinicians such as sex or gender contribute to the report of pain (18–21). “Gender” refers to the societal definition of characteristics for each sex and consists of beliefs of proper behaviors including pain behaviors. “Sex” refers to biological sex (20, 22). In Western societies, the stereotypical male gender role is characteristically stoic and tries to impress women by their capability to tolerate pain, whereas the female role displays higher sensitivity to pain to induce protective behaviors in men (19). Characteristics of observers or providers can impact the experience of pain (22–25). For instance, Aslaksen et al. (25) indicated that, compared to males tested by a male experimenter, male participants who were tested by a female experimenter reported lower pain. The status of the experimenter/clinician, like the expertise, appearance, and professionalism, is another characteristic that may influence the report of pain or placebo effects (22, 26–31). For instance, Mercer et al. (32) reported that patients perceived clinicians wearing laboratory coats as more professional, whereas clinicians with informal clothes were rated less professional, compared to clinicians with laboratory clothes (29, 32, 33).

### Experimenter or Clinician Nonverbal Behaviors

NB is present in almost all human interactions and conveys information that may modulate the verbal message. NB is behavior without a linguistic component (34) and refers to expression of thoughts and feelings through nonverbal expressions (35). NBs can be automatic (36) and may gain priority when there is an incongruity between nonverbal and verbal information (37). NB is divided into positive (NBs that convey a positive emotion, attitude, or relationship) and negative (NBs that convey a negative emotion, attitude, or relationship); and into micro-level (e.g., smiling, leaning forward, hand movement, eye contact, tone of voice, and body gesture) and macro-level behaviors (i.e., a collection of micro-level behaviors that conveys a psychological meaning such as dominance, confidence, or warmth) (38). NB contributes to building of relationships, provides signs about unspoken thoughts and emotions, and strengthens or contradicts verbal information (39). Also, the perception of NBs can be nonconscious and automatic (35, 40–43). Research suggests that NBs of experimenters/clinicians can modulate pain, and placebo/nocebo effects [e.g., Refs. (22, 44)]. For instance, Ambady and Gray (40) demonstrated that clinician’s negative NBs, such as lack of smiling, a larger distance from patients, and looking away from them, contributed to decreased cognitive (focused attention and level of consciousness) and physical functioning (walking across a room and getting up from a chair) of patients. Another study indicated that negative NBs of clinicians impacted patient’s health outcome as keeping a larger distance, and not looking at patients decreased the satisfaction with the consultation (45).

Thus, the characteristics and NBs of the experimenter/clinician can have consequences for health (3) and a review is therefore warranted. This review investigated 1a) whether experimenters’/clinicians’ sex can impact pain and placebo/nocebo effects, 1b) whether the status of experimenters/clinicians influences pain and placebo/nocebo effects, and 2) whether experimenter/clinician NBs affect pain and placebo/nocebo effects.

## Methods

### Search Procedure

Searches in the PubMed, EmBase, and ISI databases (Web of Knowledge) were conducted until September 10, 2018. **Table 1** shows the list of Boolean term combinations that were used to search in each database.

**Table 1:** Search terms used for the database search.

	AND	OR
“Nonverbal”	“placebo”	“nocebo”
“Nonverbal”	“pain”	
“Nonverbal”	“hyperalgesia”	“analgesia”
“Contextual factor”	“placebo”	“nocebo”
“Contextual factor”	“pain”	
“Contextual factor”	“hyperalgesia”	“analgesia”
“Situational factor”	‘‘placebo’’	‘‘nocebo’’
“Situational factor”	“pain”	
“Situational factor”	“hyperalgesia”	“analgesia”
“Context”	“placebo”	‘‘Nocebo’’
“Context”	“pain”	
“Context”	“hyperalgesia”	“analgesia”
“Subtle cues”	“placebo”	‘‘nocebo’’
“Subtle cues”	“pain”	
“Subtle cues”	“hyperalgesia”	“analgesia”
“Nonspecific factors”	“placebo”	“nocebo”
“Nonspecific factors”	“pain”	
“Nonspecific factors”	“hyperalgesia”	“analgesia”
“Experimenter sex”	“placebo”	“nocebo”
“Experimenter sex”	“pain”	
“Experimenter sex”	“hyperalgesia”	“analgesia”
“Experimenter gender”	“placebo”	“nocebo”
“Experimenter gender”	“pain”	
“Experimenter gender”	“hyperalgesia”	“analgesia”
“Physician sex”	‘‘placebo’’	‘‘nocebo’’
“Physician sex”	“pain”	
“Physician sex”	“hyperalgesia”	“analgesia”
“Physician gender”	“placebo”	‘‘nocebo’’
“Physician gender”	“pain”	
“Physician gender”	“hyperalgesia”	“analgesia”
“Clinician sex”	“placebo”	“nocebo”
“Clinician sex”	“pain”	
“Clinician sex”	“hyperalgesia”	“analgesia”
“Clinician gender”	“placebo”	“nocebo”
“Clinician gender”	“pain”	
“Clinician gender”	“hyperalgesia”	“analgesia”
“Provider gender”	“placebo”	“nocebo”
“Provider gender”	“pain”	
“Provider gender”	“hyperalgesia”	“analgesia”
“Clinician sex”	“placebo”	“nocebo”
“Clinician sex”	“pain”	
“Clinician sex”	“hyperalgesia”	“analgesia”
“Experimenter style”	“placebo”	“nocebo”
“Experimenter style”	“pain”	
“Experimenter style”	“hyperalgesia”	“analgesia”
“Experimenter status”	“placebo”	“nocebo”
“Experimenter status”	“pain”	
“Experimenter status”	“hyperalgesia”	“analgesia”
“Experimenter characteristic”	“placebo”	“nocebo”
“Experimenter characteristic”	“pain”	
“Experimenter characteristic”	“hyperalgesia”	“analgesia”
“Physician status”	“placebo”	“nocebo”
“Physician status”	“pain”	
“Physician status”	“hyperalgesia”	“analgesia”
“Physician style”	“placebo”	“nocebo”
“Physician style”	“pain”	
“Physician style”	“hyperalgesia”	“analgesia”
“Physician characteristic”	“placebo”	“nocebo”
“Physician characteristic”	“pain”	
“Physician characteristic”	“hyperalgesia”	“analgesia”

### Data Extraction

**Figure 1:**
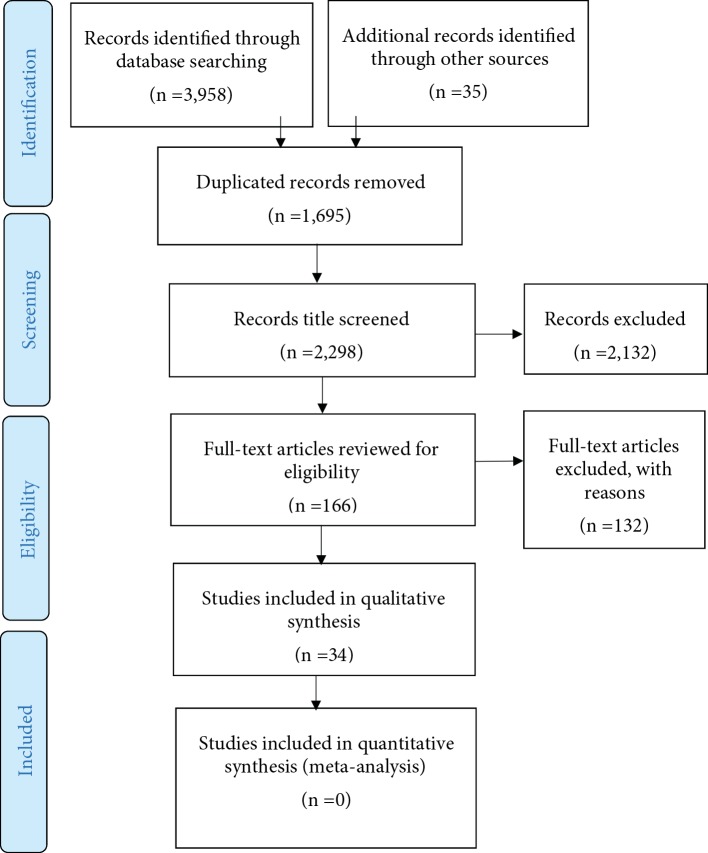
PRISMA flow diagram of steps taken in this review.

Data were extracted by the first author (HD). The second author (MF) checked the extracted data. The searches resulted in 3,958 hits. Only experimental (i.e., a causal manipulation following a random assignment in an experiment or a control group), quasi-experimental (i.e., a manipulation without *a priori* random assignment), and correlational (i.e., a non-experimental method to measure the relationship between variables) studies that investigated the contribution of characteristics and/or NBs of experimenters/clinicians to placebo, nocebo, and pain were included. Studies with both humans and animals were included and the primary target outcomes were pain report and pain behavior (e.g., pain intensity, sensitivity, threshold, duration, tolerance, unpleasantness, and pain medication use). The secondary target outcomes were symptom severity, improvement rate, mood, quality of life, and treatment expectation. A placebo response was defined as the difference between a group or condition where placebo treatment was administrated with information that the placebo was a painkiller, and a natural history control group or condition where no treatment was provided. Studies were also included if equal amounts of medication were administrated to all participants/patients, but where different types of information (verbal and/or nonverbal) about the drug were presented to different conditions and groups. Studies that reported a placebo response only as the difference between a pretest and a posttest in the same group were excluded. Studies that reported the effects of contextual factors such as group or family membership (e.g., the role of NBs of mothers on children reports of pain), race and ethnicity (e.g., the effects of black experimenter’s sex), etc., without distinction from other characteristic of experimenters/clinicians, were excluded. There were no restrictions regarding the target population of included studies. As the terms “Sex” and “Gender” are inconsistently used in studies, both terms were entered in searches, even though the present review focuses on the effects of sex. There is not a review protocol, but a list of the excluded studies is available by contacting the first author (HD) (**Figure 1**).

In line with previous studies [e.g., Refs. (38, 40)], positive NBs were defined as leaning forward, keeping less distance to the participant or patient, more body gestures, a friendly and warm voice, frequent eye contact, nodding, and smiling. Negative NBs were defined as leaning backward, increased distance to the participant/patient, less body gestures, a cold and flat tone of voice, looking away, and frowning. Thirty-four studies (20 experimental, 11 quasi-experimental, and 3 correlational studies) that reported the effect of experimenters/clinicians characteristics and/or NBs on placebo/nocebo effects or pain were included. Included studies were classified in two tables on the basis of the relativeness to whether characteristics (sex and status) (20 studies, **Table 2**) or NBs (11 studies, **Table 3**) of the experimenter/clinician. Additionally, three studies were included in both tables as they had investigated both NBs and characteristics of experimenters/clinicians. Studies were classified according to design, number of participants, sample (healthy participants, patients, or animals), type of provider (clinician or experimenter), characteristics (**Table 2**) or NB (**Table 3**), target outcome, and the result.

**Table 2:** Studies investigating the role of the experimenter/clinician characteristics (sex and status).

Study	Design	N (Female)	Sample	Type of provider	Characteristics	Target outcome	Result
Egbert et al. (46)	Between subjects	97 (63)	Abdominal surgery patients	Clinicians	Status (confidence)	Narcotic usage, physical and emotional status, postoperative pain intensity	Confident clinicians induced less postoperative narcotics and a better physical and emotional condition.
Otto and Dougher (47)	Between subjects	80 (40)	Healthy participants	Experimenters (2 M and 2 F)	Experimenter sex	Pain intensity	No significant effect of experimenter sex
Levine and De Simone (19)	Mixed design	68 (33)	Healthy participants	Experimenters (1 M and 1 F)	Experimenter sex	Pain intensity	Female experimenters induced lower pain intensity in male subjects.
Feine et al. (48)	Between subjects	20 (20)	Healthy participants	Experimenters (1 F and 1 M)	Experimenter sex	Pain intensity	No significant effect
Bush et al. (49)	Between subjects	47 (24)	Orofacial pain patients	Experimenters (1 F and 1 M)	Experimenter sex	Pain intensity	No significant effect
Fillingim et al. (50)	Between subjects	209 (117)	Healthy participants	Experimenters (1 M and 1 F)	Experimenter and participant sex	Pain thresholds	Female experimenters induced higher pain thresholds in both male and female subjects.
Carter et al. (51)	Between subjects	80 (40)	Healthy participants	Experimenters (not reported)	Experimenter sex	Pain tolerance, electrocardiogram	Female experimenters induced lower electromyogram activity and higher pain tolerance in male and female subjects.
Kállai et al. (22)	Mixed design	160 (80)	Healthy participants	Experimenters (4 M and 4 F)	Status (professional or informal) Experimenter sex	Pain threshold, pain intensity, pain tolerance	Professional experimenters generated higher pain tolerance in participants. Experimenters induced higher pain tolerance in an opposite sex participant. Female experimenters induced higher pain intensity in male and female subjects.
Thorn et al. (52)	Correlation	219 (129)	Healthy participants	Experimenters (1 M and 1 F)	Experimenter sex	Pain intensity and tolerance	No significant effects.
Essick et al. (53)	Between subjects	34 (17)	Healthy participants	Experimenters (1 M and 1 F)	Experimenter sex	Pain sensitivity	No significant effect.
Gijsbers and Nicholson (54)	Between subjects	64 (32)	Healthy participants	Experimenters (1 M and 1 F)	Experimenter gender	Pain threshold	Female experimenters induced higher pain thresholds in male subjects.
Campbell et al. (26)	Between subjects	117 (117)	Healthy participants	Experimenters	Status (university professor or a graduate assistant)	Blood pressure response, pain tolerance, and unpleasantness	Professors generated higher blood pressure responsivity, higher pain tolerance, and lower pain unpleasantness in subjects.
Aslaksen et al. (25)	Mixed design	64 (32)	Healthy participants	Experimenters (3 M and 3 F)	Experimenter and participants gender	Pain intensity, heart rate, and skin conductance	Female experimenters induced lower pain reports in male subjects.
Williams et al. (55)	Correlation	70 (41)	Low back pain patients	Clinicians	Status (a clinician or an assistant)	Pain intensity recollection after an injection	Clinicians generated recalled-pain ratings in patients that correlated the ratings presented following the procedure. Assistants generated higher pain ratings than their original ratings.
Aslaksen and Flaten (56)	Within subjects	63 (32)	Healthy participants	Experimenters (4 M and 4 F)	Experimenter gender	Pain intensity, placebo, stress, arousal, mood	Male experimenters induced higher placebo responses in male subjects.
Kaptchuk et al. (57)	Between subjects	262 (199)	Patients with IBS	Clinicians	Status (a placebo acupuncture augmented by confidence VS a placebo acupuncture alone—limited—and a waiting list)	Placebo, global improvement, adequate relief, symptom severity, quality of life	Confident clinicians generated higher global improvement, adequate relief of symptoms, better quality of life, and lower symptom severity scores in participants.
Weimer et al. (58)	Between subjects	64 (32)	Healthy participants	Experimenters (1 M and 1 F)	Experimenter sex	Placebo, nausea	No interaction between experimenter sex and placebo responses.
Vigil et al. (59)	Mixed design	352 (169)	Healthy participants	Experimenters (7 M and 7 F and 1 T)	Experimenter gender (and a transgendered experimenter with a feminine status), participant gender	Pain intensity	Male experimenters induced lower pain intensity in both male and female subjects. Transgendered experimenters induced higher pain intensity in female subjects.
Vigil and Alcock (60)	Correlation	199 (86)	Pain patients	Clinicians (not reported)	Experimenter gender	Pain intensity	Both sex patients presented higher pain levels to female clinicians.
Sorge et al. (61)	Mixed design	–	Mice	Experimenters (4 F and 4 M)	Experimenter sex	Facial grimacing as a pain behavior	Male experimenters induced less pain behaviors and more pain inhibition in rodents.
Vigil et al. (62)	Mixed design	132 (132)	Menstrual Pain patients	Clinicians (12 M and 7 F)	Clinician sex	Pain tolerance	Male clinicians induced higher pain tolerance in female patients.
Modić Stanke and Ivanec (27)	Mixed design	96 (69)	Healthy participants	Experimenters	Status (either a professor or a student)	Pain threshold	Professors generated higher pain thresholds in participants. Status influenced males more.
Howe et al. (44)	Between subjects	160 (80)	Healthy Participants	Experimenters	Status (high or low competence)	Placebo and nocebo effects (positive and negative expectations)	Competent experimenters enhanced the effects of positive expectations about a placebo cream on allergic responses.

Just for studies in which the effects of experimenter’s/clinician’s sex were investigated, the number of providers is presented; however, some studies have not reported this number. F, females; M, Males; T, transgendered; IBS, irritable bowel syndrome.

**Table 3:** Studies investigating the role of the experimenter/clinician nonverbal behaviors.

Study	Design	N (Female)	Sample	Type of provider	Nonverbal behavior	Target outcome	Result
Egbert et al (46)*	Between subjects	97 (63)	Abdominal surgery patients	Clinicians	Enthusiasm and rapport	Narcotic usage, physical and emotional status, postoperative pain intensity	Enthusiastic clinicians induced less postoperative narcotics and a better physical and emotional condition.
Gryll and Katahn (63)*	Mixed design	160 (75)	Dental patients	Clinicians	Warm or neutral behavior of clinicians	Pain intensity, state anxiety	Enthusiastic messages generated lower pain intensity, less anxiety, and higher placebo effects in subjects.
Brown et al. (64)	Mixed design	101 (49)	Healthy participants	Experimenters	*Active support* (reassuring, encouraging, more understanding, more concern, more eye contact and body gestures), *passive supports* (small talk, few comments, trying to distract the participant, no eye contact or body gestures)	Pain intensity	Active and passive support of providers generated lower pain intensity compared to alone condition.
Kaptchuk et al. (57)	Between subjects	262 (199)	Patients with IBS	Clinicians	A placebo acupuncture *augmented* by a warm and friendly manner, active listening and empathy VS a placebo acupuncture alone (*limited*) and a waiting list	Placebo, global improvement, adequate relief, symptom severity, quality of life	Warm and friendly manner of clinicians generated higher global improvement, adequate relief of symptoms, better quality of life, and lower symptom severity scores in participants.
Verheul et al. (65)	Between subjects	30 (30)	Patients with menstrual pain	Clinicians	*Warm and empathic communication* (directing gaze and body posture toward participants) VS *cold and formal communication* (directing gaze and body posture away from participants).	State anxiety, affective state and outcome expectancies	Warm and empathic communication combined with positive expectations led to lower state anxiety. Affect-oriented communication style of providers influenced the positive and negative affects of participants.
Modi´c Stanke and Ivanec (66)	Mixed design	48 (48)	Healthy participants	Experimenters	Physical distance	Pain reports	No significant effects for the physical distance of unfamiliar observers on participants’ pain reports.
Bohns and Wiltermuth (67)	Between subjects	89 (44)	Healthy participants	Experimenters	Personal space and tone of voice	Pain threshold	Preserving the personal space and speaking softly led to higher pain thresholds.
Valentini et al. (68)	Within subjects	27 (12)	Healthy participants	Experimenters	Observation of *facial expressions* with different emotional content (neutral, pain, and happy)	Pain intensity, placebo effect	Facial expressions (especially happy faces) boosted the placebo analgesia.
Ruben and Hall (69)	Within subjects	95 (55)	Healthy participants	Experimenters	*Nonverbally supportive provider* (friendly voice, leaning forward, open body posture, eye contact, nodding, and smiling) VS *nonverbally unsupportive provider* (rarely looking to camera, looking down frequently, folding arms, leaning back, distancing, cold tone of voice).	Judging the intensity of pain in the target (either with a supportive or a unsupportive clinician)	Nonverbally supportive experimenters generated more valid impressions of pain ratings and judges had higher accuracy in their pain assessment when viewing participants tested by supportive clinicians than subjects tested by nonverbally unsupportive clinicians.
Ruben et al. (70)	Mixed design	205 (129)	Healthy participants	Experimenters	*High nonverbal support*: leaning forward, eye contact, nodding, smiling, gesturing, and using a warm voice tone; *low nonverbal support*: poor eye contact, looking down frequently, folded arms, leaning back, distancing from participants, and a cold voice tone.	Pain tolerance, pain intensity	Nonverbally supportive experimenters induced higher pain tolerance and a reduction in the expressed pain in participants, compared to less nonverbally supportive clinicians that induced lower pain tolerance in participants.
Czerniak et al. (71)	Between subjects	122 (46)	Healthy participants	Experimenters	*Performance status*: scenario A: sitting, minimal eye contact, lack of tactile interaction like shaking hands; scenario B: sitting and standing, frequent eye contact, tactile interaction.	Pain threshold and tolerance	Scenario B resulted in an increase in pain threshold compared to scenario A.
Howe et al. (44)	Between subjects	160 (80)	Healthy participants	Experimenters	*High warm communication* (more eye contact, more smile, less interpersonal distance), VS *low warm communication* (less eye contact, no smiles, more interpersonal distance).	Placebo and nocebo effects (positive and negative expectations)	Warmer experimenters enhanced the effects of positive expectations about a placebo cream on allergic responses. Less warm experimenters negated the effects of expectations about a placebo cream on allergic response.
Van Osch et al. (72)	Between subjects	293 (293)	Patients with menstrual pain	Clinicians	*Positive affect-oriented communication*: warm, emphatic; VS negative: cold, formal	Anxiety, mood, expectations, satisfaction	Positive affect-oriented communication reduced anxiety, negative mood, and increased satisfaction, whereas negative communication negated positive expectations and led to higher negative mood and anxiety.
He et al. (73)	Between subjects	89 (67)	Healthy participants	Experimenters	*Communication status*: *Warm*: strong tone, animated facial expressions, frequent eye contact, expressive hand gestures, open posture. *Neutral*: monotonous voice, neural facial expression, less frequent hand gesture, minimal eye contact.	Expectation of treatment outcome, motor coordination	Warm experimenters induced higher positive expectations of treatment outcome, more improvement in performance, and better balance and coordination, compared to neutral experimenters that induced lower positive expectations.

*The procedure of these studies are not well described, but it seems a “social interaction” factor that referred to a warm or neutral behavior of the provider was at play, and also a “pill administration message” factor that reffered to the content of the information about the pill. However, this last factor is confusing and we are not completely sure about what was actually manipulated.

### Bias Risk Assessment

In order to represent trustable outcomes, systematic reviews should acknowledge a number of risk of biases (74). Although there is not a protocol review, the aims of this study did not change throughout the study and the risk of reporting bias (i.e., changing the aims according to the nature of obtained findings) was avoided (74). To avoid the risk of evidence selection bias (lack of access to all of the accessible information), the references and citation lists (in google scholar) of all included studies were manually searched and studies that fulfilled the inclusion criteria were entered. Although there is no consensus on what tool to assess the risk of bias in different types of studies, the Cochrane risk of bias tool was used to evaluate the risk of bias in experimental studies that used random assignment and a control group (75). This tool provides a categorized qualitative judgment about the level of risk (high, low, or unknown) across a number of bias types, and includes random sequence generation (i.e., concerning randomization and random sampling), allocation concealment (i.e., hiding the nature of exposure and control groups from participants and personnel), blinding of participants and personnel, blinding of outcome assessment (e.g., the level of objectiveness in outcome assessments), incomplete outcome data (i.e., concerning the missing data and dropouts), selective reporting (i.e., reported and unreported findings), and other biases [for comprehensive information, see Ref. (75)]. To evaluate the risk of bias in quasi-experimental and correlational studies, the Risk of Bias Assessment tool for Non-randomized Studies (RoBANS) was used. RoBANS can be used to assess all study types except for randomized control trials and contains six domains for the risk of bias, which are the selection of participants, confounding variables (i.e., lack of clear distinction between dependent and independent variables), the measurement of exposure (e.g., reliability of measures and scales used), the blinding of outcome assessments, incomplete outcome data, and selective outcome reporting. RoBANS is compatible with the Cochrane risk of bias tool and has a same qualitative judgment procedure [for more information, see Ref. (76)].

Using the Cochrane risk of bias assessment tool for experimental studies (75) and RoBANS for quasi-experimental and correlational studies (76), the risk of bias of the individual studies was judged by both authors and the second author (MF) synchronized the results in two tables (**Table 5** for Cochrane risk of bias assessment; and **Table 6** for the RoBANS; see the results).

## Results

A total of 34 studies were identified: 20 on the role characteristics, 11 on the role of NBs, and 3 studies on the role of both characteristics and NBs of the experimenters/clinicians.

### Experimenter/Clinician Characteristics

#### Experimenter/Clinician Sex and the Participants’ Pain

A total of 15 studies investigated whether the sex of the experimenter/clinician affected the pain report of research participants: Six studies showed a main effect of experimenter sex: three studies showed that male experimenters induced lower pain intensities than females did (22, 59, 60), and Sorge et al. (61) showed that male experimenters induced less pain behaviors and more pain inhibition in rodents. On the other hand, two studies reported that female experimenters induced lower pain intensities than males (50, 51). Nine studies reported no main effect for the sex of the experimenter/clinician (19, 25, 47–49, 52–54, 62) (**Table 2**).

Ten of these 15 studies investigated the interaction of experimenter and subject sex: Three studies showed that, compared to male experimenters, female experimenters induced higher pain thresholds (54), lower pain intensities (19, 25), and marginally significant lower pain unpleasantness (25) in male subjects. Two studies reported that, compared to female experimenters, male experimenters induced higher pain tolerance in female subjects (22, 62). On the other hand, five studies did not find a significant interaction of experimenter/clinician sex and participant sex on pain report (47, 50–53). The remaining four studies (48, 49, 59, 60) did not use subject sex as a dependent variable and thus could not investigate the interaction of experimenter/clinician sex and participant/patient sex. One study was on animals and was not relevant in this context (61) (**Table 2**).

In sum, there is no reliable tendency for a main effect of experimenter sex on pain. However, there is some evidence of an interactive effect, as 5 of 10 studies show that the experimenter induced less pain in a subject of the opposite sex (19, 22, 25, 54, 62) (**Table 2**).

#### Experimenter/Clinician Sex and Placebo/Nocebo Effects

Two studies investigated the role of experimenter/clinician sex on placebo/nocebo effects: Aslaksen and Flaten reported that, compared to female experimenters, male experimenters contributed to higher placebo responses in male subjects (56). However, Weimer et al. (58) who studied the effects of ginger and a placebo on nausea, reported no interaction between experimenter sex and placebo responses (**Table 2**; for a review, see **Table 4**).

**Table 4:** An overview of the effects of experimenter/clinician sex on pain and placebo effects.

	Study	Sex effect	Target	Finding
1	Otto and Dougher (47)	No effects	Pain	–
2	Feine et al. (48)	No effects	Pain	–
3	Bush et al. (49)	No effects	Pain	–
4	Thorn et al. (52)	No effects	Pain	–
5	Essick et al. (53)	No effects	Pain	–
6	Weimer et al. (58)	No effects	Placebo	–
7	Levine and De Simone (19)	Interaction effect	Pain	Female experimenters induced lower pain reports in males.
8	Gijsbers and Nicholson (54)	Interaction effect	Pain	Female experimenters induced lower pain reports in males.
9	Aslaksen et al. (25)	Interaction effect	Pain	Female experimenters induced lower pain reports in males.
10	Vigil et al. (62)	Interaction effect	Pain	Male experimenters induced lower pain reports in females.
11	Aslaksen and Flaten (56)	Interaction effect	Placebo	Male experimenters induced lower pain reports in males.
12*	Kállai et al. (22)	Interaction effect	Pain	Opposite sex experimenters induced lower pain reports. (i.e., females reported higher pain tolerance to male experimenters)
12	Kállai et al. (22)	Main effect	Pain	Female experimenters induced higher pain intensity report in both sex subjects.
13	Vigil et al. (59)	Main effect	Pain	Male experimenters induced lower pain reports in both sex subjects.
14	Vigil and Alcock (60)	Main effect	Pain	Female clinicians generated higher pain reports in both sex patients.
15	Sorge et al. (61)	Main effect	Pain	Male experimenters induced lower pain expressions in mice.
16	Carter et al. (51)	Main effect	Pain	Female experimenters induced lower pain reports in both sex subjects.
17	Fillingim et al. (50)	Main effect	Pain	Female experimenters induced lower pain reports in both sex subjects.

*The study of Kállai et al. (22) has reported both interaction and main effects. Therefore, this study is considered twice.

In sum, there is no reliable tendency for the impact of experimenter sex on placebo effects (**Table 2**).

#### Experimenter/Clinician Status and Participants’ Pain

Five studies investigated the effects of experimenter/clinician status on pain reports of research participants: Three studies showed that compared to lower professional status (a student or an assistant), higher-status (e.g., a faculty member or a professor) experimenters generated higher pain thresholds (27) and tolerance (22, 26) and lower pain unpleasantness (26). Williams and colleagues (55) reported that in comparison with research assistants, clinicians contributed to more accurate pain ratings (i.e., recollections of pain intensity following a surgery, correlated with pain ratings presented at the time of surgery) in low back pain patients. Also, Egbert et al. (46) reported that confident clinicians had patients with less usage of narcotics and in a better physical and emotional state than patients of less confident clinicians (**Table 2**).

In sum, all the five studies showed that higher professional status and higher confidence of experimenters/clinicians led to lower pain reports (22, 26, 27), more accurate pain ratings (55), and better physical and emotional state (46). No studies reported other effects of experimenter/clinician status on pain (**Table 2**).

#### Experimenter/Clinician Status and Placebo/Nocebo Effects

Two studies investigated the effects of the status of experimenters/clinicians on placebo/nocebo effects: Kaptchuk and colleagues (57) showed that, compared to less confident practitioners, more confident clinicians induced higher symptom relief, higher scores on a global improvement scale, and less symptom severity in patients with irritable bowel syndrome (IBS). Howe et al. (44) reported that competent experimenters (who made no mistakes throughout the experiment) induced higher placebo effects (**Table 2**).

To sum up, two studies revealed that confidence and competence status of experimenters/clinicians generated higher placebo effects (44, 57). No studies reported other effects of experimenter/clinician status on placebo effects (**Table 2**).

### Nonverbal Behaviors

#### Experimenter/Clinician Nonverbal Behaviors and Participants’ Pain

Seven studies investigated the effects of experimenters/clinicians NBs on the pain of research participants: Ruben et al. (70) showed that, compared to clinicians with negative NBs, clinicians with positive NBs induced higher pain tolerance and less pain expressions. In another study, Ruben and colleagues (69) showed that clinicians with positive NBs generated more accurate pain ratings (i.e., consistency between expressions of pain by subjects and judgments about pain ratings by observers), compared to clinicians with negative NBs. Czerniak et al. (71) showed that a clinician with restricted movements, minimal eye contact, more typing, and lack of tactile interaction such as shaking hands induced lower pain thresholds in participants. In comparison, a clinician that had more eye contact, more body movements, shook hands with patients, and touched the patients through the examination had patients who displayed higher pain thresholds. Bohns and Wiltermuth (67) showed that preserving the physical space (not getting too close to the participants) and speaking softly led to higher pain thresholds, whereas lack of preserving the physical space and speaking loudly led to lower pain thresholds. On the other hand, Egbert et al. (46) reported that patients who were visited by a more enthusiastic clinician had less usage of narcotics and their surgeons considered them in a better physical and emotional condition and ready to discharge from hospital. Brown et al. (64) reported no significant difference between the pain reports of participants who received “active support” (including more eye contact and body gestures) and “passive support” (lack of eye contact or body gestures). However, both groups had lower pain reports than the “alone” (undergoing the experiment alone) group, suggesting that the NBs of the clinician reduced pain report. Modić Stanke and Ivanec (66), on the other hand, reported that closer physical distance of observers from participants did not have any significant effect on the pain report of participants (**Table 3**).

In sum, six of seven studies concluded that positive NBs of experimenters/clinicians resulted in lower pain reports (64, 67, 70, 71), more accurate pain ratings (69), and less narcotic use and better physical and emotional state (46), whereas negative NBs led to higher pain reports and lower pain tolerance (67, 70, 71). On the other hand, one study failed to find a significant effect of experimenters/clinicians NB (66) (**Table 3**).

#### Experimenter/Clinician Nonverbal Behaviors and Placebo/Nocebo Effects

Seven studies investigated the effects of experimenters/clinicians NBs on placebo/nocebo effects: Gryll and Katahn (63) found that enthusiastic messages of clinicians generated higher placebo responses and less anxiety in patients that received dental treatment. Another study showed that, compared to the limited interaction (5-min duration, and a very small talk about the sham injection), an augmented communication style (45-min interaction, including a warm and friendly manner) of clinicians resulted in lower pain intensity reports, higher symptom relief, higher scores on a global improvement scale, and less symptom severity (57); whereas limited communication style of clinicians led to higher pain severity reports, lower scores on the global improvement scale, and less symptom relief and higher symptom severity reports by patients (57). Furthermore, compared to a cold communication style (i.e., directing gaze and body posture away from participants and no empathic remarks), a warm communication style (i.e., gazing at the patient, welcoming in a friendly manner, an open body posture, and adding empathic remarks) of clinicians resulted in positive expectations (expectations of shorter pain duration), decreases in anxiety and negative mood, and higher treatment satisfaction in women with menstrual pain (65, 72). A cold communication style of clinicians resulted in higher anxiety levels and expectations of longer pain duration in patients (65, 72) (**Table 3**).

He et al. (73) showed that, compared to a neutral communication style (speaking in a monotone voice, neutral facial expressions, less hand movements, and less eye contact), clinicians with a positive communication style (including strong tone of voice, dynamic facial expressions, eye contact, hand gestures, and open body postures) induced stronger positive expectations in a coordination and balance test and believed their coordination ability improved more (**Table 3**).

Howe et al. (44) showed that, compared to a “low-warmth” clinician who used minimal eye contact, no smiles, and had more distance from participant, a “high-warmth” clinician who used more eye contact, more smiles, and had closer distance enhanced the impact of positive expectations about the effects of an inert cream on their allergic responses, and lowered the allergic reactions. Valentini et al. (68) showed that compared to neutral facial expressions, participants had higher placebo effects when they were exposed to more facial expressions with emotional contents. Interestingly, higher placebo effects were reported when participants observed smiling faces (68) (**Table 3**).

To sum up, all seven studies reported that positive NBs of experimenters/clinicians enhanced the placebo effects and negative NBs lowered placebo effects or increased nocebo effects (44, 57, 63, 65, 68, 72, 73). There were no studies that indicated other effects of NBs (**Table 3**).

### Risk of Bias Assessment

Of the 20 experimental studies, 19 had low risk of bias in random sequence generation, 16 had low risk of bias in allocation concealment, 12 had unclear risk of bias in blinding of participants and personnel, 16 had low risk of bias in blinding of outcome assessment, 18 had low risk of incomplete outcome data, and 19 had low risk of selective reporting bias (**Table 5**).

Of the 14 quasi-experimental and correlational studies, 10 studies had low risk of bias in selection of participants, 13 had low risk of confounding variables, 7 had low risk of bias in measuring the exposure, 9 had unclear risk of bias in blinding of outcome assessments, 10 had low risk of incomplete outcome data, and 8 studies had unclear risk of bias in selectively reporting the outcomes (**Table 6**).

**Table 5:** Cochrane Risk of bias assessment for experimental studies of the effects of experimenters/clinicians characteristics and non-verbal behaviors on pain and placebo effects.

				1	2	3	4	5	6	7	
Egbert et al. (46) Levine and De Simone (19) Carter et al. (51) Brown et al. (64) Kallai et al. (22) Gijsbers and Nicholson (54) Kaptchuk et al. (57) Verheul et al. (65) Stanke and Ivanec (66) Weimer et al. (58) Bohns and Wiltermuth (67) Vigil et al. (59) Sorge et al (61) Stanke and Ivanec (27) Ruben and Hall (69) Czerniak et al. (71) Ruben et al. (70) Van Osch et al. (72) Howe et al. (44) He et al. (73)				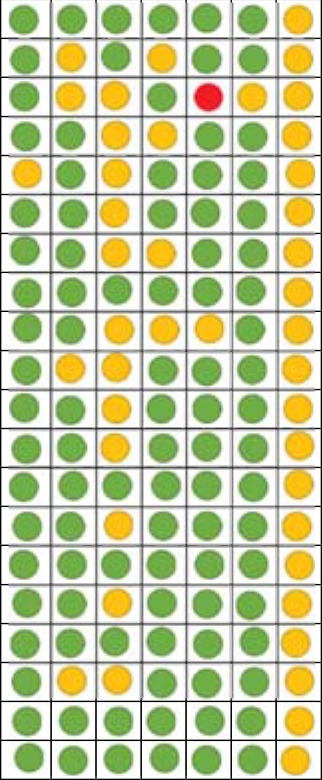							
	Key										
	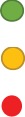		Low risk of bias Unclear risk of bias High risk of bias								

**Numbers’ definition:**

1. Random sequence generation (selection bias)

2. Allocation concealment (selection bias)

3. Blinding of participants and personnel (performace bias)

4. Blinding of outcome assessment (detection bias)

5. Incomplete outcome data (attrition bias)

6. Selective reporting (reporting bias)

7. Other bias

**Table 6:** Risk of Bias Assessment for quasi-experimental and correlational studies (RoBANS) of the effects of experimenters/clinicians characteristics and non-verbal behaviors on pain and placebo effects.

				1	2	3	4	5	6	
Gryll and Katahn (63) Otto et al. (47) Feine et al. (48) Bush et al. (49) Fillingim et al. (50) Thorn et al. (52) Essick et al. (53) Campbell et al. (26) Aslaksen et al. (25) Williams et al. (55) Aslaksen and Flaten (56) Vigil and Alcock (60) Valentini et al. (68) Vigil et al. (62)				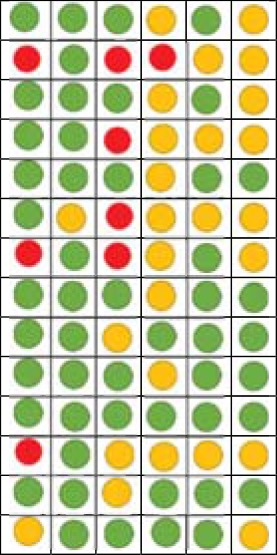						
	Key									
	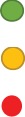		Low risk of bias Unclear risk of bias High risk of bias							
**Numbers’ definition:**										

1. Selection of participants

2. Confounding variables

3. Measurement of exposure

4. Blinding of outcome assessments

5. Incomplete outcome data

6. Selective outcome reporting

## Discussion

Several findings emerged: 1) Five of 10 studies showed an interactive effect of experimenters and participants’ sex such that experimenters induced less pain in a participant of the opposite sex. There was, on the other hand, no reliable main effect of experimenter sex on the reports of pain. 2) All five studies showed that experimenters/clinicians of a higher status and confidence induced less pain in participants or had patients who had less narcotic usage. 3) Two of two studies showed that experimenters of a high status induced larger placebo effects. 4) Six of seven studies showed that positive NBs induced less pain. 5) All seven studies showed that positive NBs induced larger placebo responses. 6) All seven studies showed that negative NBs induced lower placebo responses or higher nocebo effects.

### The Role of Experimenter/Clinician Sex on Pain and Placebo Effects

Five of 10 studies showed that participants reported lower pain when tested by an experimenter of the opposite sex. Thus, the tendency of an interaction of experimenter/clinician sex and the sex of the participant must be considered with caution. Previous studies have suggested that this tendency can be related to the experimenter gender role rather than to biological factors. For instance, Aslaksen et al. (25) showed that although female experimenters contributed to lower pain report in male subjects, the female experimenters did not have a significant effect on the heart rate variability of the subjects. Thus, the impact of the pain stimulus on autonomic nervous system activity was the same in both male and female participants. This suggests that the lower reported pain in males tested by a female was a reporting bias. In the same line, Flaten et al. (2) showed that female experimenters induced lower pain reports in male participants and concluded that this could be due to a response bias in males, so they were trying to impress female experimenters by reporting lower pain. Interestingly, Gijsbers and Nicholson (54) showed that by exaggerating the gender-related appearance and behaviors of female experimenters, the hypoalgesic effect of female experimenters on male subjects can be enlarged.

Two studies (22, 62) showed that male experimenter/clinicians induced lower pain reports in female subjects. This finding contradicts the conventional gender role assumptions that assumed a helpless state for females, in which they display higher pain to induce male protection. Kállai et al. (22) showed that females reported lower pain to male experimenters and concluded that females, as well as males, try to impress opposite sex experimenters by their ability to tolerate pain longer. This can be due to changes in the female gender role in contemporary societies in which more authority and power are granted for females.

A second explanation attributes the hypoalgesic effects of male experimenters on female subjects to the physiological aspects of females. Vigil et al. (62) tested two groups of high- and low-fertility females by male and female experimenters and showed that, compared to females who were tested by a female experimenter, high-fertility females who were tested by a male experimenter reported lower pain. This finding suggests that physiological factors can contribute to the lower pain reports of female subjects to male experimenters/clinicians. Also, this finding can partially explain why some studies [e.g., Ref. (25)] failed to observe a hypoalgesic effect of male experimenters on female subjects.

There was no reliable effect of experimenter/clinician sex on placebo analgesia (56, 58).

### The Role of Experimenter/Clinician Status on Pain and Placebo Effects

Five studies showed that higher status of experimenters/clinicians generated lower pain reports. Campbell et al. (26) demonstrated that subjects displayed higher blood pressure reactivity and pain tolerance to higher-status experimenters and concluded that increased blood pressure stimulated pressure receptors in the vasculature that also modulate the perception of pain (77–84). The stress induced by the higher-status experimenters may therefore lead to lower pain reports (26).

Two studies demonstrated that higher status of the experimenters/clinicians induced larger placebo effects. Howe et al. (44) showed that competent clinicians enhanced the effects of positive expectations and reduced subject’s allergic responses. They suggested that outcome expectations, that are underlying factors for the placebo and nocebo effects, can be modulated by the warmth and competence of clinicians. Notably, Howe et al. (44) studied the effects of low-competence characteristics of clinicians on negative expectations, and did not observe a significant effect on negative expectations.

### The Role of Experimenter/Clinician Nonverbal Behaviors on Pain and Placebo Effects

Six studies showed that positive NBs of experimenters/clinicians induced lower pain reports, and three studies showed that negative NBs resulted in higher pain reports. Pain is recognized as a stressor and most of painful situations induce stress and negative emotions (54, 85). Negative emotions and stress can increase the experience of pain [e.g., Refs. (56, 85)], whereas providing information about the forthcoming intervention and outcomes of a treatment may reduce the stress and negative emotions. As there can be uncertainty about the outcome of interventions (54, 85), participants/patients might use as much of accessible information as possible to gain knowledge about the efficacy of the intervention. NBs of experimenters/clinicians can be a substantial source of information for participants/patients (36, 69, 70). In this line, Ambady and Gray (40) showed that positive NBs of clinicians (e.g., facial expressiveness, nodding, and smiling) were associated with long-term improvements in cognitive and physical functioning of elderly patients. Previous studies have shown that clients can perceive the expectations of their providers [e.g., Refs. (36, 86)]. As interpersonal expectations are mostly communicated through NBs [e.g., Ref. (38)], positive NBs of experimenters/clinicians can be interpreted as a sign of satisfactory functioning or results and lead to decrease in negative emotions and subsequently lower pain reports, whereas negative NBs can be assumed as a sign of negative forthcoming results and lead to higher pain reports. In this line, Egbert et al. (46) showed that patients who were exposed to enthusiastic clinicians were in a better emotional state, and Gryll and Katahn (63) showed that enthusiasm by clinicians reduced negative emotions.

Seven studies showed that positive NBs of experimenters/clinicians induced higher placebo effects, whereas negative NBs led to lower placebo effects and higher nocebo hyperalgesia. To explain the modulatory effects of NBs on placebo and nocebo effects, a similar perspective is taken. NBs may have a confirmatory (or contradictory) role for verbal information that is used to induce positive outcome expectations and placebo effects. So, positive NBs may have an additive value for the verbal information, e.g., that a tablet is a powerful pain killer, and negative NBs may contradict the verbal information and diminish the induction of placebo effects. In this line, Howe et al. (44) showed that positive NBs of clinicians enhanced the impact of positive expectations about the effects of an inert cream on allergic responses; and He et al. (73) showed that positive NBs of clinicians induced stronger positive expectations in a coordination and balance test. Expectations may also contribute to the modulation of emotions and stress. For instance, Verheul et al. (65) and Van Osch et al. (72) reported that positive NBs of clinicians enhanced positive outcome expectancies and reduced the state anxiety and negative mood, whereas negative NBs resulted in higher anxiety levels and expectations of longer pain duration.

Therefore, NBs may have an additive value for the role of verbal information in modulation of expectations, negative emotions, and stress, and hence lead to changes in amplitudes of placebo or nocebo effects. Several studies have reported failure to elicit a placebo effect [e.g., Refs. (58, 87)]. Uncontrolled NBs of experimenters/clinicians may partially account for such diversity in findings.

## Conclusion

This qualitative review documented the contribution of experimenters/clinicians’ sex, status, and NBs, as three factors capable of altering the perception of pain, and amplitude of placebo/nocebo effects and responses.

Sex, status, and NBs of experimenters/clinicians are interwoven in every laboratory and clinical setting and the present review shows that these factors can influence research results. The failure to control for the effects of characteristics and NBs of experimenters/clinicians can explain why placebo studies occasionally yield inconsistent or variable findings [e.g., Refs. (58, 87, 88)], or why the reliability of pain measurement is limited and doubted [e.g., Ref. (25)]. To gain a deeper understanding of the effects of such nonspecific factors, this review emphasizes the need to further investigate the contribution of characteristics and NBs of experimenters/clinicians in pain and placebo effects.

### Recommendations for Future Research

Prospective investigations are encouraged to address the following gaps in current literature; first, to our knowledge, just two studies have investigated the separate effects of different NBs on pain and placebo effects (68, 73). Thus, future studies should specify what specific NBs (facial expressions, eye contact, nodding, physical distance, tone of voice, or body postures) that have the strongest impact on pain and placebo/nocebo effects; He et al. (73) showed that compared to physical distance and body posture, facial expressions and tone of voice had stronger effects on placebo effects. However, this finding should be replicated especially in prospective pain studies. Second, the interaction of NBs and sex of providers and subjects should be investigated to see whether NBs of experimenters can modulate the effects of sex or vice versa. Only one study has studied this and reported that positive NBs of experimenters induced lower pain reports in male subjects than in female subjects (70). Third, future studies should suggest how to control for the effects of NBs in research on pain and other symptoms. Indeed, this can only be achieved if we have more knowledge about the effects of each specific NB on pain or other symptoms. Fourth, studies could consider the effects of other genders (e.g., transgendered experimenters) on the experience of pain; to our knowledge, only one study has addressed this (59) and showed that compared to a male or female experimenter who acted in accordance with their sex, a biological male who acted in a feminine way induced higher pain reports in female subjects. Fifth, there might be an interaction between experimenters/clinician’s sex and their status. Several studies have reported that for example, male providers were considered more credible (87); their status influenced male subjects more (27); male clinicians who were reputed for their expertise were more preferred over female clinicians; and female clinicians who were reputed for their interpersonal skills were preferred more by patients (30). The possible interaction of the status and the sex of the experimenters/clinicians should be taken into account to determine whether status can modulate the effects of sex or vice versa. According to our searches, only Kállai et al. (22) have tested both sex and status systematically, but unfortunately have not reported the interaction of sex and status of the experimenters. Lastly, the underlying mechanisms (e.g., expectations and emotions) of the effects of NBs and characteristics of experimenters/clinicians on pain and placebo effects are still unclear and should be investigated. More knowledge of these factors would be highly relevant in the training of health personnel.

## Limitations

The present study contains a number of limitations that should be noted here. First is the qualitative nature of this study that hinders the generality of findings. Second is the heterogeneity of keywords used in different studies, which made it difficult to gain access to all related studies and may have caused to miss a few studies; however, to prevent this, several Boolean searches were conducted and also the reference and citation lists of included studies were checked. Third is the interpretation of the findings on the interaction of the experimenters’/clinicians’ sex and subjects’ sex. Of the included studies, five studies showed an interaction, and five studies did not find an interaction. Therefore, the findings on the interaction of the experimenter/clinician and participant’s sex should be interpreted with caution. Fourth is the problem of confounding in some findings such as investigating the provider status and NBs simultaneously and without differentiation as in Kaptchuk et al. (57); or lack of clarity in methodological procedures such as absence of differentiation in providers’ sex and status as in Campbell et al. [Ref. (26) or (87)]; or lack of differentiation between verbal and nonverbal components as in Gryll and Katahn (63). Such deficiencies limit the drawing of straightforward conclusions. Additionally, this systematic review did not comprise a review protocol, but authors tried to precisely characterize the scientific nature of this systematic review by determining *a priori* question and the procedure relevant to the questions.

## Author Contributions

MF planned the study. HD searched and extracted the articles and MF screened them. Both authors significantly contributed to the analyses of results, drafting of the manuscript, and preparation of the final draft.

## Funding

The present research was funded by the Norwegian University of Science and Technology (NTNU).

## Conflict of Interest Statement

The authors declare that the research was conducted in the absence of any commercial or financial relationships that could be construed as a potential conflict of interest.
